# The Effectiveness and Safety of *Abelmoschus manihot* in Treating IgA Nephropathy: A Systematic Review and Meta-Analysis

**DOI:** 10.1155/2022/9730753

**Published:** 2022-10-05

**Authors:** Qi Jia, Jing Guo, Yuzi Cai, Weijun Huang, Zebing Zhu, Chenhui Xia, Keting Guo, Hongcai Shang, Yuning Liu, Weijing Liu

**Affiliations:** ^1^Department of Neurology, Dongzhimen Hospital Affiliated to Beijing University of Chinese Medicine, Beijing, China; ^2^Key Laboratory of Chinese Internal Medicine of Ministry of Education and Beijing, Dongzhimen Hospital Affiliated to Beijing University of Chinese Medicine, Beijing, China; ^3^Renal Research Institution of Beijing University of Chinese Medicine, Dongzhimen Hospital Affiliated to Beijing University of Chinese Medicine, Beijing, China

## Abstract

**Introduction:**

IgA nephropathy (IgAN) is a common issue. In China, *Abelmoschus manihot* (AM) is widely used in the treatment of IgAN. However, their combined effectiveness and safety for this purpose have not yet been explored. AM is an effective medicine for treating IgAN. This meta-analysis aimed to evaluate the effectiveness of AM for IgAN.

**Materials and Methods:**

The Cochrane Library, PubMed, EMBASE, Allied and Complementary Medicine Database (AMED), Chinese Biomedical Literature Database (CBM), Chinese National Knowledge Infrastructure Database (CNKI), Chinese Science and Technique Journals Database (VIP), and the Wanfang Database were searched from their inceptions to June 2021. Random clinical trials (RCTs) comparing the effects of AM treatment in patients with IgAN were included. The study evaluated the efficacy or effectiveness of AM for IgAN and had clear outcome data, such as total effectiveness rate or proteinuria.

**Results:**

A total of 11 RCTs with 850 participants were included in this meta-analysis. The results of the meta-analysis showed that, compared with that of the conventional therapy alone, being combined with conventional treatment was significantly more effective for the total efficacy rate (OR = 4.33; 95% CI = 2.66, 7.04; *P* < 0.00001) and proteinuria (MD = −0.41 g/24 h; 95% CI = −0.44, −0.38; *P* < 0.00001) but had no effect on serum creatinine (Scr) (MD = −2.23 *μ*mol/L; 95% CI = −5.90, 1.45; *P*=0.24), eGFR (MD = −0.45 mL/min·1.73 m2; 95% CI = −1.24, 2.13; *P*=0.60), Bun (MD = −0.22 mmol/L; 95% CI = −0.59, 0.14; *P*=0.23), systolic blood pressure (MD = −0.04 mmHg; 95% CI = −2.59, 2.51; *P*=0.98), diastolic blood pressure (MD = −0.34 mmHg, 95% CI = −1.65, 2.33; *P*=0.74), systolic blood pressure (MD = −0.04 mmHg, 95% CI = −2.59, 2.51; *P*=0.98), or serum albumin (MD = 1.70 g/L, 95% CI = −1.06, 4.45; *P*=0.23).

**Conclusions:**

AM provided additional benefits to proteinuria individuals with IgAN. However, due to the high clinical heterogeneity and small sample size of the included trials, future studies should conduct more rigorous RCTs on the clinical efficacy and safety of AM and RCTs with a larger sample size involving multicenters.

## 1. Introduction

IgAN is the most common worldwide primary glomerular disease, which leads to chronic kidney disease (CKD) and even end-stage renal disease (ESRD). It can occur at all ages, but the peak occurrence is at 20–40 years. There is a significant regional difference in the incidence of IgAN, which is significantly higher in Asia than in other regions. A total of 13,519 kidney biopsy data in China showed that IgAN accounted for 45% of primary glomerular diseases. Up to 50% of IgAN patients can gradually enter ESRD within 20–25 years, which suggests that it is important to actively treat IgAN and control its progression [1–3].

A variety of treatments have attempted to release the burden on the kidneys and reduce the high risk of kidney failure in IgAN patients. The clinical manifestations are varied, among which the presence of microscopic hematuria and proteinuria are the most common [4]. The Guide to Prognosis of Kidney Disease Improving Global Outcome (KDIGO) suggests that renin-angiotensin system inhibitors are used for patients with IgAN consisting of persistent proteinuria ≥0.5 g/d, and renin-angiotensin system inhibitors plus corticosteroid treatment are used for IgAN patients with proteinuria ≥1 g/d (2012). At present, there are no specific drugs for the treatment of IgAN and no drugs for the treatment of IgAN have been approved by Food and Drug Administration (FDA). This kind of nephropathy is mainly treated with drugs, such as ARBs/ACEI, to alleviate symptoms but often fails to meet the treatment needs of patients. Due to the limited treatment methods currently available, it is necessary to conduct a novel, effective, and safe treatment for IgAN.

The flower of *Abelmoschus manihot* (Linn) Medicus (family *Malvaceae*), namely, Flos *A. manihot*, was used to treat inflammatory diseases in China [5]. Huangkui capsule (HKC), purified from AM, gained approval from China's State Food and Drug Administration (Z19990040) for the treatment of chronic nephritis in 1999 [6]. Several studies have shown that HKC improved renal inflammation in CKD, including nephrotic syndrome, membranous nephropathy, IgAN, and DN effectively used in clinical diagnosis [7–9]. Recently, increasing clinical evidence in China has been suggested that HKC is the safe and effective dose of 7.5 g/kg/day can reduce microurinary albumin (micro-UAlb) in IgAN patients [10, 11] and that its therapeutic action may be concerned with immunological reaction, inflammation, renal fibrosis, and renal tubular epithelial injury [12].

However, previous studies have not been sufficiently systematic. Therefore, we conducted a meta-analysis of randomized controlled trials to determine whether or not AM is beneficial to patients with IgAN. To evaluate the effect of AM, being combined with standard ARBs/ACEIs was used in the experimental group, and standard ARBs/ACEIs alone was administered in the control group. Our objective was to evaluate the benefits and potential harms of AM for treating IgAN.

## 2. Methods

### 2.1. Protocol and Registration

This systematic review and meta-analysis were conducted by the order of the PRISMA [13]. It is available on the International Prospective Register of Systematic Review (PROSPERO), with a registration number CRD42018104427.

### 2.2. Inclusion and Exclusion Criteria

Inclusion criteria were as follows: (1) the patients of every single study were diagnosed as IgAN by renal biopsy; (2) the participants of the treatment groups were given 2.5 g of AM three times a day; (3) the conventional therapy was angiotensin-II receptor blockers/angiotensin-converting enzyme inhibitors alone, AM combined with conventional treatment was treatment groups; (4) the studies evaluated the efficacy or effectiveness of AM for IgAN and has clear outcome data, such as total effectiveness rate or proteinuria; and (5) selected RCTs for the treatment of IgAN.

Exclusion criteria were as follows: (1) the study subjects did not rule out secondary IgAN, such as lupus nephritis, purpuric nephritis, or hepatitis-associated nephropathy; (2) the study subjects did not rule out factors affecting proteinuria, such as fever, infection, or heart failure; (3) the test group and/or control group used hormonal therapy; and (4) the full text could not be obtained.

### 2.3. Search Strategies

We searched the following sources for the identification of trials: the Cochrane Library, PubMed, EMBASE, Allied and Complementary Medicine Database (AMED), Chinese Biomedical Literature Database (CBM), Chinese National Knowledge Infrastructure Database (CNKI), Chinese Science and Technique Journals Database (VIP), and the Wanfang Database. Databases of ongoing trials were also searched. Search terms for PubMed (free words search) were as follows: (huangkui OR ambrette OR abelmoschus OR *Abelmoschus manihot*) and (IgAN OR mesangial proliferative glomerulonephritis OR glomerular disease). A different search strategy was applied for Chinese and foreign language databases. Conference abstracts were searched manually. All abovementioned databases were searched from the available date of inception until the latest issue (6). No other restrictions were performed, and the free-text strategy and Medical Subject Headings (MeSH) terms were conducted in the term-searching process. The searching language in Chinese, English, and Japanese was slightly changed based on the situation of adaptation to different databases.

### 2.4. Data Selection

Two reviewers (Qi Jia and Jing Guo) independently screened the literature, extracted the data, and cross-checked each other. If there was any disagreement, a third party was consulted to assist in the judgment. Any lacking information was investigated by contacting the authors and by requesting for the missing information. When screening the literature, the title and abstract were first read. After excluding unrelated literature, the reviewers read the full text to determine whether it would be included in the present study or not. The data extraction content primarily included the following: basic information for the study, comprising research titles; first author; published journal and time; baseline characteristics of the study, including the number of samples and the age, gender, and disease status of the patients in each group; specific details of the intervention; follow-up time; risk of bias; key elements of the evaluation; outcome indicators; and outcome measurement data of interest. We have contacted the authors of the trial to get more information from papers. Two of the authors (Qi Jia and Jing Guo) evaluated the risk of bias of each trial independently in accordance with the CONSORT-CHM. All criteria were referred to from the Cochrane guidelines. There were three categories of results: “low risk of bias,” “unclear risk of bias,” and “high risk of bias.”

### 2.5. Data Extraction

Two authors (Qi Jia and Jing Guo) used data extraction tables designed before the beginning of the literature retrieval to extract the following data: publication information, sample size, age, proteinuria, serum creatinine, intervention group, control group, treatment duration, and outcome. The effects of AM were measured by proteinuria and the total effective rate. The characteristics of the trials are summarized in Table 1.

### 2.6. Outcome Measures

Meta-analysis was performed using RevMan 5.3. The dichotomous data adopted the odds ratio (OR) as the effect index, the measurement data used the mean difference (MD) as the effect index, and each effect quantity was given its point estimate and 95% CI. As the outcomes of this meta-analysis, proteinuria, Scr, eGFR, blood pressure, and serum albumin were presented as MD, while the effect rate was presented as OR.

The heterogeneity between the included studies was analyzed using the *χ*^2^ test, and I^2^ was used to quantitatively determine the size of heterogeneity. *I*^2^ values of 25%, 50%, and 75% corresponded to low, medium, and high levels of heterogeneity, respectively. If there was heterogeneity in an acceptable range between the results of each study, a fixed-effects model was used for meta-analysis; if there was statistical heterogeneity among the results, further analysis of heterogeneity sources excluded the effects of significant clinical heterogeneity. After that, a meta-analysis was performed using a random-effects model. Significant clinical heterogeneity was treated by subgroup analysis, sensitivity analysis, or only descriptive analysis [24]. Funnel plots were interpreted to report biases. The prespecified subgroup analysis was performed according to the difference in treatment duration.

### 2.7. Methodological Quality

Two reviewers (Qi Jia and Jing Guo) independently assessed the methodological quality of the studies according to the Cochrane Collaboration's risk of bias tool. If there was any disagreement, a third party was consulted to assist in the judgment. The assessment included the following seven components: (1) random sequence generation; (2) allocation concealment; (3) blinding; (4) assessor blinding; (5) incomplete outcome data; (6) selective reporting; and (7) other sources of bias.

## 3. Results

### 3.1. Study Selection

**Figure 1:**
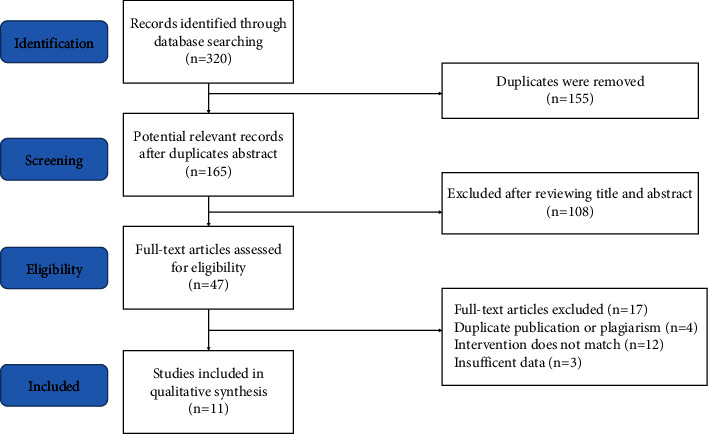
The flowchart of the study-screening process.

We identified 320 relevant articles from six different electronic databases, and after screening through layers, 155 studies were excluded for the reason of duplication. Next, 108 records were removed for improper titles and abstracts, and 47 publications were further ruled out after full-text analysis for the following reasons: four publications were duplicates or were plagiarized; interventions of ten studies were not matched; three studies had insufficient data; and two studies were nonrandomized clinical trials. Eventually, 11 articles [11, 14–23] were included in this systematic review and meta-analysis. The flowchart of the study screening process in the meta-analysis is presented in Figure 1.

### 3.2. Characteristics of Eligible Studies

There were 850 patients in this study (experimental group: 432 patients and control group: 418 patients). For the included articles, all of them except eight studies used ACEI as the control treatment [17, 19, 22], while ARB was used in the remaining studies. All included trials, except for four studies, recruited participants with a normal Scr. Of the five articles, three studies [11, 19, 20] recruited patients with eGFR >60 ml/min; one study [22] (24) involved patients with Scr <264 *μ*mol/L; and one study [17] received participants with Scr <350 *μ*mol/L. The proteinuria of all enrolled participants was less than 3.5 g/d. Regarding the selection of treatment periods, 1 study [19] reported changes after 16 weeks, 4 studies [14–16, 18] detected changes after 12 weeks, 4 studies [17, 20–22] were limited to eight weeks, and 2 studies [11, 23] only identified changes after 24 weeks. The primary characteristics of the trials are summarized in Table 1.

### 3.3. Risk of Bias

**Figure 2:**
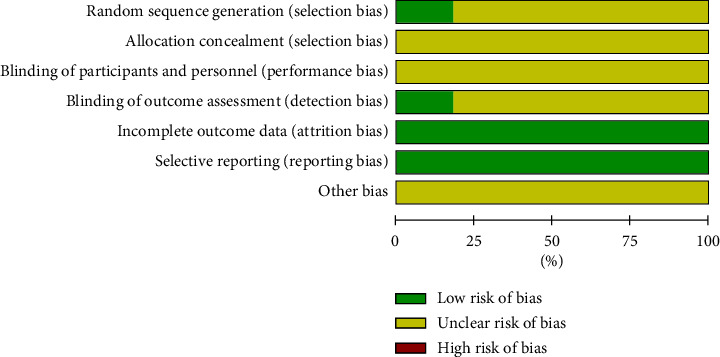
Risk of bias assessment.

**Figure 3:**
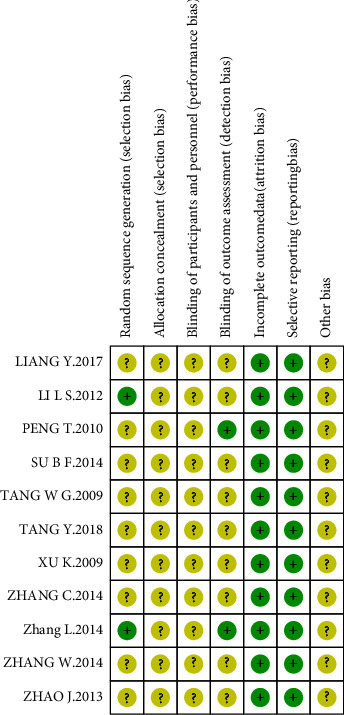
Risk of bias in individual studies.

We carried out the risk of bias assessment based on the information retrieved from the trials. The details of the risk biases are summarized in Figure 2 and Figure 3.

#### 3.3.1. Allocation

All included studies mentioned randomization. However, only two [11, 14] of them reported the specific methodology (i.e., a random number table) and (low risk of bias). Allocation concealment was not mentioned in any of the studies. Accordingly, all trials were at unclear risk of selection bias.

#### 3.3.2. Blinding

None of the articles illustrated that experimenters or participants were blind to the experimental conditions; therefore, the trials were assessed at a high risk of performance bias. None of the trials reported blinding of outcome assessment; therefore, the trials were at unclear risk of detection bias.

#### 3.3.3. Incomplete Outcome Data

All included clinical trials reported having no missing outcome data and included all participants in the data analyses. Therefore, we assessed these trials at low risk of bias.

#### 3.3.4. Selective Reporting

The risk of bias in selective reporting was high, as none of the nine studies published their protocols and lack of data on adverse events and health-related quality of life outcomes. The other two trials [14, 21] reported adverse events (unclear risk of bias).

#### 3.3.5. Other Potential Sources of Bias

All included trials showed free of other factors that could put them at risk of bias. We classified the included trials at low risk of other biases.

### 3.4. The Effects of Interventions

#### 3.4.1. Effective Rate

**Figure 4:**
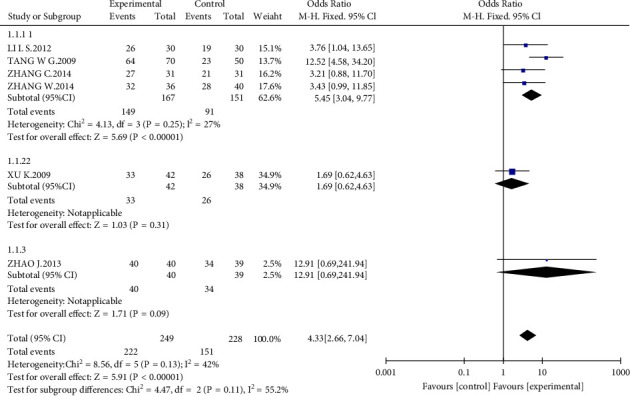
Forest plot of the effects of interventions.

Six studies [14, 17, 19–22] with a total of 477 patients reported clinical curative efficiency. The efficiency rates of the four studies [14, 17, 20, 21] were defined as follows: basic remission: 24-hour urine protein measurement 0.2 g, disappearance of highly active red blood cells, and/or normal renal function. 24-hour urine protein measurement 0.2 g, 50% less than before treatment, and/or 3 high-powered red blood cells, normal or minimally normal renal function, 15% deviation from normal value; improvement: 24-hour urine protein quantification that is 25%–50% less than before therapy and/or contains no more than 5 high-powered red blood cells, as well as normal or enhanced renal function; and ineffective: no improvement or decline in the aforementioned indexes. No change or deterioration in the aforementioned indicators indicates that the kidney function is normal or improving. The efficiency of Xu et al.'s study [19] is defined as follows: complete remission: no urine protein, no urine red blood cells, normal renal function. Basic remission is defined as a reduction of more than 50% in urine protein and red blood cells, as well as normal or nearly normal renal function. Urine protein and red blood cell reductions of more than 25%, normal or improved renal function and ineffective: no change in urine protein, red blood cells, or renal function tests. The efficiency of Zhao's study [22] is defined as follows: complete remission: no urine protein, no urine red blood cells, normal renal function. Basic remission is defined as a reduction of more than 50% in urine protein and red blood cells, as well as normal or nearly normal renal function. Urine protein and red blood cell reductions of more than 25%, normal or improved renal function and ineffective: no change in urine protein, red blood cells, or renal function tests. The heterogeneity test (*I*^2^ = 42%, *P* = 0.13) indicated moderate statistical heterogeneity between studies, so we applied a fixed-effects model to measure the combined odds ratio (OR = 4.33) and 95% CI as 4.43 (2.66, 7.04, *P* < 0.00001), indicating a statistically significant difference between groups. We performed a subgroup analysis in terms of different efficiency criteria. A total of 240 patients in four studies [14, 17, 20, 21] are shown in Figure 4, AM could significantly improve the therapeutic effect of ACEI/ARBs for IgAN (MD = 5.45; 95% CI, 3.04 to 9.77; *P* < 0.00001).

#### 3.4.2. Proteinuria

**Figure 5:**
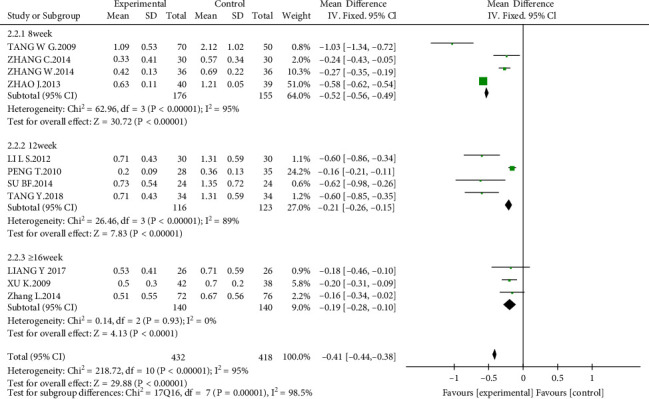
Effect of AM with ACEIs/ARBs therapy on proteinuria.

All studies included 850 participants who assessed proteinuria. Proteinuria occurred at a lower rate among people in the combined group compared with those in the control group at the end of treatment (MD = −0.41 g/24 h; 95% CI −0.44 to −0.38; *P* < 0.00001) (Figure 5). We performed a subgroup analysis in terms of different treatment periods. Interestingly, we found that the MD became smaller with the prolonging of the treatment period. A total of 331 patients in four studies (eight weeks) showed that 24-h proteinuria in the experimental group was significantly lower than that of the control group (MD = −0.50 g/24 h; 95% CI, −0.74 to −0.26; *P* < 0.00001). There were four articles over 12 weeks (*n* = 239) demonstrating that AM had better downregulated effect in 24-h proteinuria than that in the control group (MD = −0.48 g/24 h; 95% CI, −0.78 to −0.17; *P* < 0.0001). Additionally, the other three studies (*n* = 280) in which the treatment period was greater than 16 weeks reported that AM could significantly reduce the level of 24-h proteinuria more than that in the control group (MD = −0.19 g/24 h; 95% CI, −0.28 to 0.10; *P* < 0.00001).

#### 3.4.3. Kidney Function

**Figure 6:**
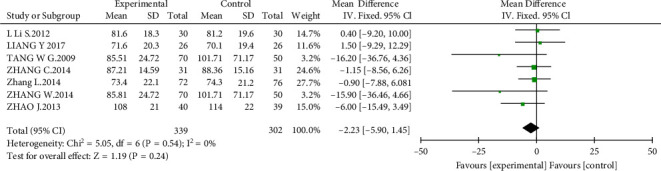
Effect of AM with ACEIs/ARBs therapy on Scr.

**Figure 7:**
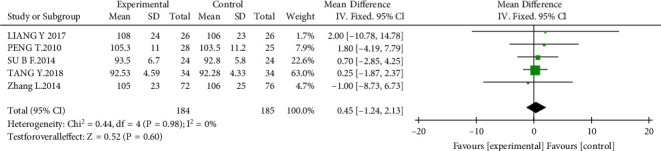
Effect of AM with ACEIs/ARBs therapy on eGFR.

Kidney function was measured by estimated glomerular filtration rate (eGFR) or serum creatinine (Scr). A total of seven studies [11, 14, 17, 20–23] (*n* = 641) were included to evaluate Scr levels. The results reported that there was no significant difference in the experimental group compared with the control group (MD = −2.23 *μ*mol/L, 95% CI, −5.90 to 1.45; *P* = 0.24; Figure 6). As for eGFR, five studies [11, 15, 16, 18, 23] (*n* = 369) were included. However, the results showed no difference between the experimental group and the control group (MD = −0.45, 95% CI, −1.24 to 2.13; *P* = 0.60; Figure 7).

#### 3.4.4. Blood Urea Nitrogen (BUN)

**Figure 8:**
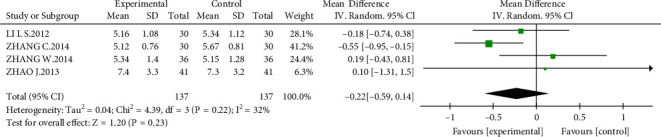
Effect of AM with ACEIs/ARBs therapy on Bun.

Four studies [14, 20–22] that recruited 274 participants were used to assess the urea nitrogen (BUN) level. However, no significant difference was observed between the AM group and the control group (MD = −0.22 mmol/L, 95% CI, −0.59 to 0.14; *P* = 0.23; Figure 8).

#### 3.4.5. Blood Pressure

**Figure 9:**
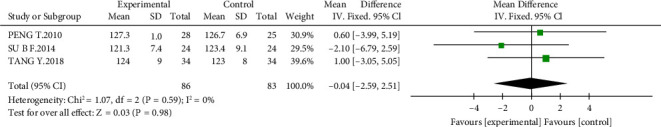
Effect of AM with ACEIs/ARBs therapy on systolic blood pressure.

**Figure 10:**
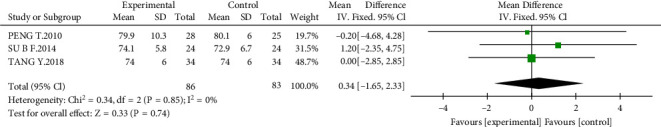
Effect of AM with ACEIs/ARBs therapy on diastolic blood pressure.

Three studies [15, 16, 18] reported the blood pressure level in 169 patients. However, no significant difference was found in systolic blood pressure (MD = −0.04 mm Hg, 95% CI, −2.59 to 2.51; *P* = 0.98) or diastolic blood pressure (MD = −0.34 mm Hg, 95% CI, −1.65 to 2.33; *P* = 0.74) at the end of the treatment or during follow-ups (Figures 9 and 10).

#### 3.4.6. Albumin

**Figure 11:**
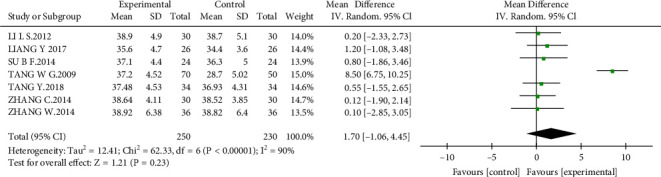
Forest plot of albumin.

There were seven studies [14, 16–18, 20, 21, 23] that reported the albumin level in 480 patients. However, albumin was not significantly changed in patients with AM compared with that in the control group at the end of the treatment or during follow-up (MD = 1.70 g/L, 95% CI, −1.06 to 4.45; *P* = 0.23; Figure 11).

### 3.5. Adverse Reactions

Adverse reactions were monitored in two studies. There was not an incident of adverse events in the study by Li et al. [14], and Zhang et al. [21] ^[18]^ showed that one person developed slight dizziness in the intervention group; in the control group, abdominal distension was found for one case, and dizziness was found in another case.

### 3.6. Publication Bias

**Figure 12:**
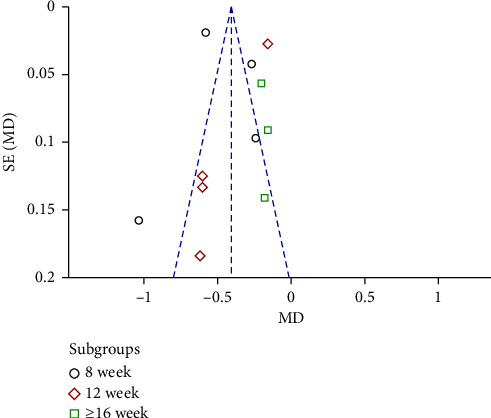
Publication-bias chart.

A funnel plot was adopted to summarize the publication bias. We found that the funnel plot was not completely symmetrical, which indicated that some publication bias was present in these studies (Figure12).

## 4. Discussion

IgAN, which is now known as slowly progress to end-stage renal disease, is the most common type of glomerulonephritis around the world [25]. Berger and Hinglais first discovered IgAN in 1968, and IgAN represents the pivotal reason for kidney failure among most populations [26]. Aberrant glycosylation of IgAN exerts an autoimmune response, which generates antiglycan antibodies. Consequent immune complex deposited in the glomerular mesangium, which activates the complement pathway, stimulates mesangial cells, and induces the secretion of cytokines, which finally results in inflammation and fibrosis. Therefore, IgAN is an autoimmune disease in which immune complexes induce renal injury [27]. However, specific and effective treatment are still lacking. Only antihypertensive drugs, such as ACEI and ARB, are useful interventions [28]. In recent studies, many Chinese medicines have been demonstrated to be effective in treating kidney disease, including IgAN [29–31]. Proteinuria is an independent risk factor for the progression of IgAN [3]. Furthermore, the prognosis for patients with IgAN is worse than that for patients with other glomerular diseases with similar proteinuria levels [32]. The results indicated that the combination of AM and RAS blockers seems to be effective and safe in further reducing proteinuria in IgAN patients.

The possible mechanisms of AM in the treatment of diabetic kidney disease (DKD) could be alleviating the early glomerular pathological changes via inhibiting Akt/mTOR/p70S6K signaling, ameliorating inflammation by the inhibition of iRhom2/TACE signaling, improving lipid disorders by enhancing PPAR*α*/*γ*, protecting ER stress and suppressing the expression of TNF-*α* and TGF-*β*1 [33–36]. Studies have applied adriamycin-inducednephropathy-inducedSprague-Dawley rats, HKC has a good effect on renal inflammation by reducing TGF-*α*, TGF-*β*1 expression, and intervening p38MAPK signaling [9, 37]. It has been confirmed that these pharmacologically active compounds, isolated from AM, have numerous beneficial biological effects. Moreover, hyperoside could protect against cisplatin-induced AKI by inhibiting oxidant response and inflammatory [38]. Myricetin can protect the kidney from cisplatin-induced toxicity partly by decreasing the number of inflammatory mediators, including TNF-*α* and IL-6 [39].

This is the first comprehensive systematic review and meta-analysis to evaluate the effects of AM on proteinuria and renal function in IgAN patients. In the present study, we reviewed 11 RCTs involving a total of 850 participants and assessed the add-on effects and safety of AM to ACEIs/ARBs in people with IgAN. None of the included trials mentioned ESRD rates, and as for eGFR, five studies showed no difference between the AM plus a RAS blocker and a RAS blocker alone. The results showed that the combination of AM and RAS blockers was associated with significant improvement in proteinuria compared with RAS blockers. The range of 24-h proteinuria included in the study was 0.5 g/24 h–3.5 g/24 h, and the HKC was suitable for people with a small amount of urine protein (i.e., the urine protein was lower than 3.5 g/24 h). The recommended dose of HKC for inclusion in the study was 2.5 g three times a day. We found that AM could significantly reduce the level of 24-h proteinuria than that in the control group when the treatment period was more than 16 weeks and the funnel plot also indicates 8 weeks trials are inconclusive, 12 weeks does not show a difference while more than 16 weeks may demonstrate the clinical difference, so we suggest the treatment duration for the future trials. We recommend clinical experimenters require patients to provide information that affects the progression of IgAN, such as ethnicity, family history, and comorbidity/risk factors in future research. The results also showed that AM may be generally well-tolerated, as an addition to RAS blockers, it did not increase the incidence of adverse events.

There were some potential limitations of our meta-analysis. First, some significant heterogeneity in these included studies was observed, which may be due to the small sample size and short treatment periods, and the nature of the disease course in the effective rate. The limitation to detecting a significant difference between the combined therapy group and the control group may exist. Second, all involved studies were conducted in different centers in China, and all involved patients were Chinese, so it is unavoidable that our meta-analysis had some regional bias.

## 5. Conclusion

Adjuvant therapy of AM with ARBs/ACEIs provided additional benefits on proteinuria in individuals with IgAN. However, a large number of RCTs will be required in the future to verify this speculation. If the positive effect of AM is confirmed by more high-quality clinical trials in the future, it may potentially become a complementary therapy for IgAN.

## Figures and Tables

**Table 1:** Basic characteristics of the included studies.

Studies	Sample size (I/C)	Age(I/C)	Proteinuria (g/24 h) (I/C)	Scr (*μ*mol/L) (I/C)	Intervention group	Control group	Treatment duration	Outcome
[14]	30/30	(32.8 ± 12.70)/(34.5 ± 11.7)	(1.48 ± 0.51)/(1.35 ± 0.63)	(87.4 ± 26.6)/(86.4 ± 25.4)	*A manihot* (2.5 g tid) plus olmesartan (20 mg/d)	Olmesartan (20 mg/d)	12w	24hUTP scr BUN ALB fib
[15]	28/25	(39.9 ± 7.9)/(41.7 ± 8.7)	(0.80 ± 0.38)/(0.78 ± 0.21)	—	*A manihot*(2.0 g tid) plus valsartan(80 mg/d)	Valsartan (80 mg/d)	12w	24hUTP eGFR SBP DBP
[16]	24/24	(34.4 ± 7.9)/(34.2 ± 8.1)	(1.85 ± 0.77)/(1.92 ± 0.66)	—	*A manihot* (2.5 g tid) plus irbesartan(150 mg/d)	Irbesartan (150 mg/d)	12w	24hUTP eGFR ALB SBP DBP
[17]	70/60	(30.2 ± 16.2)/(31.3 ± 15.4)	(2.72 ± 0.78)/(2.78 ± 0.63)	(181.62 ± 22.11)/(177.60 ± 19.13)	*A manihot* (2.5 g tid) plus lisinopril (10 mg bid)	Lisinopril (10g bid)	8w	24hUTP scr ALB CHO
[18]	34/34	(37.5 ± 3.6)/(37.2 ± 3.2)	(1.9 ± 0.8)/(1.8 ± 0.7)	—	*A manihot* (2.5 g tid) plus Irbesartan(150 mg/d)	Irbesartan (150 mg/d)	12w	24hUTP eGFR SBP DBP ALB
[19]	42/38	(36.4 ± 10.8)/(35.8 ± 12.4)	(1.8 ± 0.4)/(1.9 ± 0.3)	—	*A manihot* (2.5 g tid) plus benazepril (10 mg/d)	benazepril (10 mg/d)	16w	24hUTP RBC
[20]	30/30	—	(1.27 ± 0.73)/(0.94 ± 0.43)	(90.83 ± 15.43)/(89.23 ± 16.18)	*A manihot* (2.5 g tid) plus losartan(50 mg/d)	Losartan (50 mg/d)	8w	24hUTP scr BUN ALB
[11]	72/76	(37.2 ± 10.9)/(35.5 ± 11.0)	(1.02 ± 0.43)/(1.02 ± 0.44)	(73.26 ± 21.22)/(73.26 ± 18.56)	*A manihot* (2.5 g tid)plus losartan potassium (50 mg/d)	Losartan potassium (50 mg/d)	24w	24hUTP scr eGFR
[21]	36/36	—	(1.36 ± 0.64)/(1.38 ± 0.59)	(86.54 ± 26.35)/(86.60 ± 25.87)	*A manihot* (2.5 g tid) plus telmisartan(40 mg/d)	Telmisartan (40 mg/d)	8w	24hUTP scr BUN ALB fib
[22]	20/39	(34.0 ± 9.0)/(33.0 ± 8.0)	(2.91 ± 0.39)/(2.87 ± 0.37)	(114 ± 23)/(117 ± 24)	*A manihot* (2.5 g tid) plus benazepril(10 mg/d)	Benazepril (10 mg/d)	8w	24hUTP scr BUN MCP-1
[23]	26/26	(37.1 ± 11.7)/(38.7 ± 12.1)	—	—	A manihot(2.5 g tid) plus losartan potassium(100 mg/d)	Losartan Potassium (100 mg/d)	24w	24hUTP scr ALB eEGFR

I/C: intervention group/conventional group; Scr: serum creatinine; Bun: urea nitrogen; ALB: blood Albumin; Fib: fibrin; eGFR: estimated glomerular filtration rate; SBP: systolic blood pressure; DBP: Diastolic blood pressure; CHO: cholesterol; RBC: red blood cell; and MCP-1: human macrophage chemoattractant protein-1.

## Data Availability

Aggregate data were extracted from published studies.

## Abbreviations

AM: *Abelmoschus manihot*
IgAN: IgA nephropathy
CKD: Chronic kidney disease
ESRD: Even end-stage renal disease
KDIGO: The guide to improving global prognosis of kidney disease
FDA: Food and drug administration
RCTs: Randomized clinical trials
Bun: Urea nitrogen.
